# Haemodynamic effects of methoxyflurane versus fentanyl and placebo in hypovolaemia: a randomised, double-blind crossover study in healthy volunteers

**DOI:** 10.1016/j.bjao.2023.100204

**Published:** 2023-06-28

**Authors:** Lars Øivind Høiseth, Lars Olav Fjose, Jonny Hisdal, Marlin Comelon, Leiv Arne Rosseland, Harald Lenz

**Affiliations:** 1 Department of Anaesthesia and Intensive Care Medicine, Division of Emergencies and Critical Care, Oslo University Hospital, Oslo, Norway; 2 Institute of Clinical Medicine, University of Oslo, Oslo, Norway; 3 Norwegian Air Ambulance Foundation, Oslo, Norway; 4 Division of Pre-hospital Services, Innlandet Hospital Trust, Moelv, Norway; 5 Section of Vascular Investigations, Oslo University Hospital, Oslo, Norway; 6 Department of Research and Development, Division of Emergencies and Critical Care, Oslo University Hospital, Oslo, Norway

**Keywords:** analgesia, cardiac output, healthy volunteers, hypovolaemia, methoxyflurane

## Abstract

**Background:**

Methoxyflurane is approved for relief of moderate to severe pain in conscious adult trauma patients: it may be self-administrated and is well suited for use in austere environments. Trauma patients may sustain injuries causing occult haemorrhage compromising haemodynamic stability, and it is therefore important to elucidate whether methoxyflurane may adversely affect the haemodynamic response to hypovolaemia.

**Methods:**

In this randomised, double-blinded, placebo-controlled, three-period crossover study, inhaled methoxyflurane 3 ml, i.v. fentanyl 25 μg, and placebo were administered to 15 healthy volunteers exposed to experimental hypovolaemia in the lower body negative pressure model. The primary endpoint was the effect of treatment on changes in cardiac output, while secondary endpoints were changes in stroke volume and mean arterial pressure and time to haemodynamic decompensation during lower body negative pressure.

**Results:**

There were no statistically significant effects of treatment on the changes in cardiac output, stroke volume, or mean arterial pressure during lower body negative pressure. The time to decompensation was longer for methoxyflurane compared with fentanyl (hazard ratio 1.9; 95% confidence interval 0.4–3.4; *P*=0.010), whereas there was no significant difference to placebo (hazard ratio −1.3; 95% confidence interval −2.8 to 0.23; *P*=0.117).

**Conclusions:**

The present study does not indicate that methoxyflurane has significant adverse haemodynamic effects in conscious adults experiencing hypovolaemia.

**Clinical trial registration:**

ClinicalTrials.gov (NCT04641949) and EudraCT (2019-004144-29) https://www.clinicaltrialsregister.eu/ctr-search/trial/2019-004144-29/NO.

Haemorrhage is a major cause of morbidity and mortality in trauma patients whose painful injuries may also require analgesia.^1^ There is a concern that analgesics may blunt the compensatory responses to haemorrhage, leading to earlier haemodynamic decompensation.^2^ However, concern about haemodynamic compromise may lead to inadequate analgesia.^3^^,^^4^

Methoxyflurane is a halogenated ether that has recently been approved in Europe for the emergency relief of moderate to severe trauma pain in conscious adults, after being extensively used in Australia and New Zealand.^5^, ^6^, ^7^ It has an onset of analgesic effect of <5 min, with the effect lasting 20 min.^8^^,^^9^ As methoxyflurane is intended for use in trauma patients, it is important to explore its haemodynamic effects during hypovolaemia.

Opioids are indispensable for the treatment of severe acute pain.^10^ In normovolaemia, fentanyl causes an increase in vagal tone and a reduction in heart rate.^11^ Previous studies have investigated some aspects of the haemodynamic response to opioids in conscious humans during hypovolaemia,^12^^,^^13^ but much remains to be studied.^14^ In haemorrhagic shock, the use of ketamine instead of fentanyl has been advocated because of its sympathomimetic effect^2^ and possibly less arteriolar dilatation as found in a rodent model.^15^ In a recent study, we have demonstrated fentanyl 25 μg i.v. to be equianalgesic to 3 ml inhaled methoxyflurane in healthy volunteers.^16^

Lower body negative pressure (LBNP) is an experimental model of hypovolaemia.^17^^,^^18^ By applying negative pressure to the lower extremities and abdomen, blood is shifted to the lower body, creating central hypovolaemia. LBNP enables the response to graded hypovolaemia and its compensatory mechanisms to be studied. Typically, compensated hypovolaemia is characterised by relatively normal arterial blood pressure but is followed by an abrupt reduction in blood pressure as decompensation occurs.

The objective of the present study was to explore the effects of methoxyflurane on the haemodynamic response to LBNP compared with an equianalgesic dose of fentanyl and placebo during compensated hypovolaemia. The primary endpoint was the change in cardiac output with LBNP, with the null hypothesis being that there is no effect of methoxyflurane on cardiac output during LBNP when compared with placebo. Secondary endpoints were changes in stroke volume and mean arterial pressure (MAP). The time to haemodynamic decompensation, reflecting the ability to tolerate hypovolaemia, was also a secondary endpoint.

## Methods

The study was registered in ClinicalTrials.gov (NCT04641949) and EudraCT (2019-004144-29) and monitored by the Clinical Trials Unit at The South-Eastern Norway Regional Health Authority. The study was approved by the regional ethics committee (REC South East; https://rekportalen.no, reference no. 95320, 1 April 2020) and the Data Protection Officer at Oslo University Hospital. Before inclusion, written informed consent was obtained from all subjects.

### Participants

**Fig 1:**
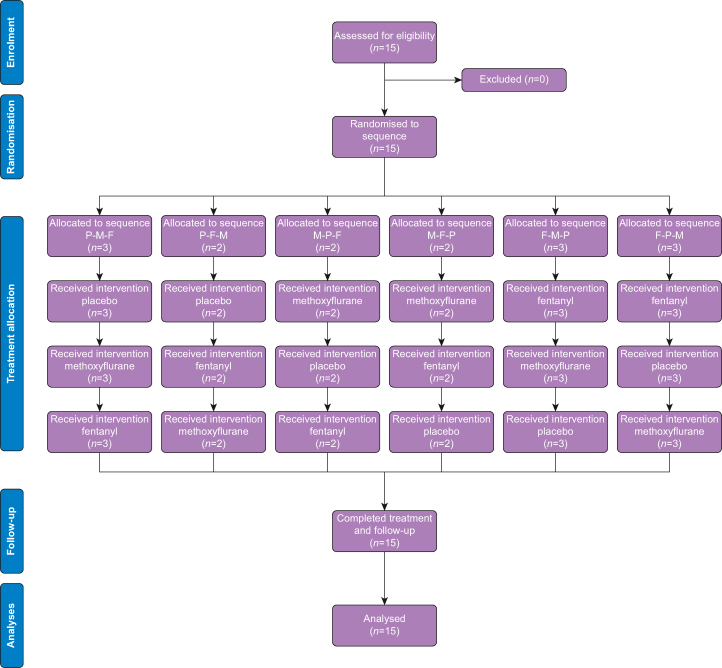
Consolidated Standards of Reporting Trials (CONSORT) diagram. F, fentanyl; P, placebo; M, methoxyflurane.

Fifteen healthy volunteers aged 18–65 yr were included. Exclusion criteria were any medical condition limiting physical exertion capacity or requiring regular medication (allergy and contraceptives excepted), chronic pain, pregnancy, substance abuse, use of pain medication or complementary medicine during the previous 2 days, or alcohol 24 h before a visit. The study was conducted according to Consolidated Standards of Reporting Trials (CONSORT) guidelines (Fig. 1).^19^^,^^20^

### Interventions

**Fig 2:**
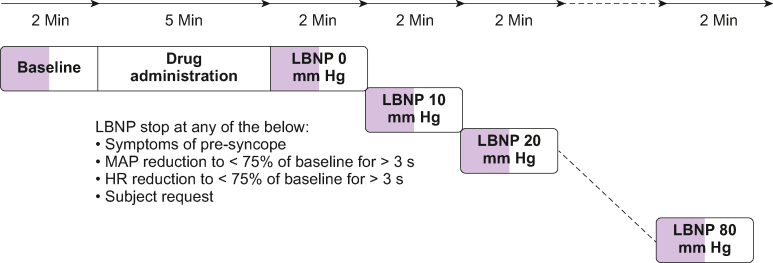
Timeline of the experimental setup for each visit. At baseline and each LBNP level, the first minute (marked purple) was allowed for stabilisation, and only data from the last minute were used for analyses. LBNP was interrupted after completing LBNP 80 or earlier if LBNP stop criteria occurred. Only data from completed LBNP levels were used for analyses, except for the calculations of time to decompensation. HR, heart rate; LBNP, lower body negative pressure; MAP, mean arterial pressure.

The subjects had three visits, with at least 2 days washout between visits. The subject was given an inhaler and an i.v. injection with one of three treatments: methoxyflurane (Visit M), fentanyl (Visit F), or placebo (Visit P). The inhaler contained methoxyflurane 99.9%, 3 ml (Penthrox®; Medical Developments NED B.V., Amsterdam, the Netherlands) at visit M, and saline 0.9%, 3 ml at visits F and P. A syringe for i.v. administration contained fentanyl 25 μg (Fentanyl Hameln 50 μg ml^−1^; Hameln Pharma GmbH, Hameln, Germany) at visit F and saline 0.9%, 0.5 ml at visits M and P. The inhaler was prepared the same day as the experiment and stored in a sealed plastic bag. Because of its distinct odour, a small amount of methoxyflurane was applied on the exterior of the inhaler to enhance blinding. The study subjects were familiarised with the laboratory setting and placed in the LBNP chamber sealed at the iliac crest.^21^ A 22G peripheral venous cannula was placed in the left antecubital vein. The subjects rested supine ∼30 min before measurements started. Baseline measurements were performed over 2 min, after which fentanyl or saline was given i.v. and the subjects breathed for 5 min through the inhaler (methoxyflurane or NaCl 0.9%) without occluding the diluter hole. Thereafter, measurements were performed for 2 min at each LBNP level with increments of 10 mm Hg, starting at 0 mmHg (Fig. 2).

### LBNP termination

The LBNP exposure was discontinued after completing LBNP 80, or earlier if decompensation occurred as defined by occurrence of any of the following: symptoms of imminent cardiovascular collapse (light-headedness, nausea, or sweating) or reduction of MAP or heart rate to <75% of baseline for >3 s. LBNP could also be stopped upon subject request for reasons other than the above.

### Measurements

A noninvasive arterial pressure waveform was obtained by the volume-clamp method around the left middle finger (Nexfin; BMEYE, Amsterdam, the Netherlands) and ascending aortic blood velocity by suprasternal Doppler (SD-50; Vingmed Ultrasound, Horten, Norway).^22^ The ventilatory frequency was measured continuously, apart from during drug inhalation, using expired CO_2_ measured by sidestream capnography (Cap10; Medlab Medizinische Diagnosegeräte GmbH, Stutensee, Germany). Arterial oxygen saturation (SpO_2_) was measured by a pulse oximeter (Masimo Radical 7; Masimo Corp., Irvine, CA, USA). Acral (fingertip) skin perfusion was measured by laser Doppler flowmetry at the left thumb (PeriFlux 4001 Master; Perimed AB, Järfälla, Sweden). These signals were sampled with an electrocardiogram (ECG; BioAmp/PowerLab; ADInstruments, Bella Vista, NSW, Australia) at 1000 Hz in LabChart 8.1.9 (ADInstruments).

### Data processing

In LabChart, values for each heartbeat were calculated delimited by the R-peaks of the ECG. MAP was calculated as the average of the arterial waveform. At Visit 1, before baseline recordings, the left ventricular outflow tract diameter was assessed using a transthoracic parasternal long-axis ultrasound image during mid-systole. The measurement was taken from inner edge to inner edge between the insertion of the leaflets. The left ventricular outflow tract area was then calculated, assuming a circular orifice and assuming it remained constant throughout this and the subsequent visits. Ascending aortic velocity–time integral×left ventricular outflow tract area gave cardiac stroke volume. Stroke volume×heart rate gave cardiac output. Ventilatory frequency and end-tidal CO_2_ (ETCO_2_) were obtained from capnography. Data were handled in R 4.05 (R Foundation for Statistical Computing, Vienna, Austria)/RStudio 1.4.1106 (RStudio, Boston, MA, USA) using the ‘tidyverse’ packages.^23^

Cerebral (ScerO_2_) oxygen saturation was measured by near infrared spectroscopy (Invos 5100C cerebral/somatic oximeter; Somanetics, Troy, MI, USA), averaged from sensors over the left and right forehead. Values were downloaded every 7–8 s and time-synchronised to the other signals in R/RStudio.

As the primary aim was to study the response to compensated hypovolaemia, haemodynamic data from the LBNP level at which decompensation occurred were discarded. At each completed LBNP level, data from the first minute were also discarded to allow for stabilisation. A trimmed mean, trimming cardiac cycles with the 10% highest and lowest values, gave one observation per completed LBNP level per visit. For time to decompensation, data from incomplete LBNP levels were included.

### Symptoms

After LBNP exposure, the subjects rated symptoms related to the visit on a verbal numerical rating scale (VNRS) from 0 to 10, where 0 was no symptom and 10 was the worst symptom imaginable. If decompensation occurred during the visit, the subjects were asked to disregard symptoms immediately related to decompensation.

### Statistics

#### Sample size

The estimated effect of LBNP on cardiac output with standard deviation (sd) was calculated from a previous experiment.^24^ A change in cardiac output of 15% is often used as a threshold for clinical significance.^25^ Using simulations, we evaluated the effect of treatment on cardiac output as 0.18 L min^−1^ for each 20 mm Hg increment in LBNP (as used in the previous study^24^) with an sd of 0.18 L min^−1^, giving a mean effect of 0.72 L min^−1^ (15% of the baseline cardiac output of 4.8 L min^−1^) at LBNP 80. When entered as an interaction term (indicating the difference in effect of LBNP) in a linear mixed regression model, 15 subjects gave a power of 0.84 to detect this difference with ⍺=0.05.

#### Randomisation and blinding

As there were six different possible treatment orders, a randomisation list was generated before the start of the study using the ‘blockrand’ package^26^ with a block size of six, assigning included subjects sequentially. One investigator (MC) kept the list and prepared the drugs but was never present during the experiments. The first author (LØH) enrolled the subjects. The subjects and investigators present during the experiments were blinded to the treatment allocation. The statistical analyses for the primary outcome were performed with dummy codes for treatment before unblinding.

#### Statistical methods

Data are mean (sd) or median (25, 75 percentiles) unless otherwise stated, after being visually assessed for normality using histograms and QQ-plots. Regression assumptions were assessed by plotting residuals *vs* predicted values. Two-sided *P*-values <0.05 were considered statistically significant.

Analyses were performed in linear mixed models (random intercept) with subject as a random effect, using the *nlme* package in R/RStudio.^27^ Mixed regression models were used to account for repeated measurements within subjects, and also because they handle missing values well. The treatment effects (explanatory variable, as factor) on the haemodynamic variables (outcome) were evaluated by treating LBNP level as a continuous explanatory variable starting at LBNP 0. To account for differences at baseline, regressions were performed on *changes* from baseline. The interactions between LBNP and treatment were considered the treatment-specific effects, as they describe the change in effect of LBNP by the treatment. The main effects of treatment describe changes from baseline to LBNP 0. To allow for a non-linear effect of LBNP, polynomial regressions up to the third degree were constructed, removing non-significant interaction effects and higher polynomials down to a minimal final model of at least a linear main effect of LBNP, the main effects of treatment and their interactions. Estimates of the effects of LBNP presented are for changes in one LBNP level of 10 mm Hg.

Estimates with confidence intervals (CI) for each drug at each LBNP level were calculated by treating LBNP levels as factors using the *glht* function of the *multcomp* package, correcting for multiple calculations within each LBNP level by the *single step* method.^28^ The time to decompensation was evaluated in a mixed Cox regression model, using the *coxme* package^29^ and *post hoc* Tukey contrasts using the *multcomp* package.^28^ Symptoms and SpO_2_ were compared by Friedman test with Nemenyi *post hoc* tests using the *PMCMRplus* package.^30^

## Results

Fifteen subjects (seven female) were included in the study with age 24 (22–28) yr, weight 73 (7.1) kg, and height 175 (8.7) cm. All subjects received the allocated treatments and were evaluated for primary and secondary outcomes. The first visit of the first subject was December 2020, and the last visit of the last subject was March 2022.

### Cardiac output

**Fig 3:**
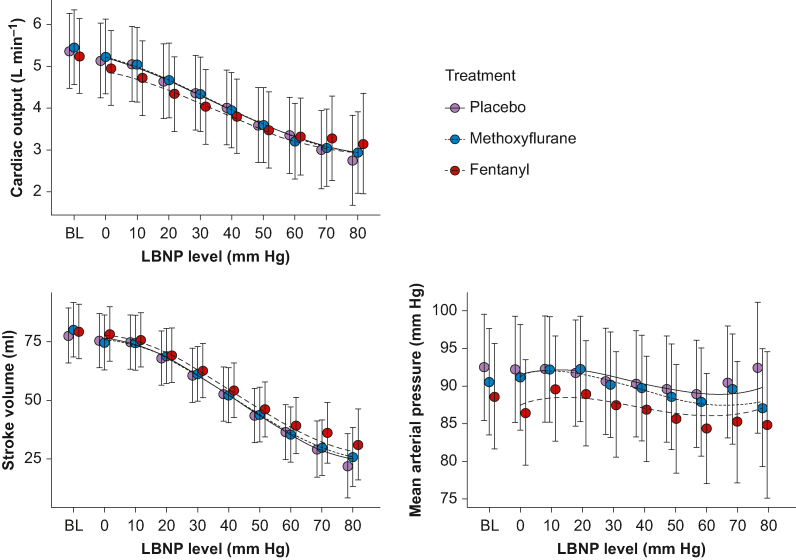
Primary and secondary outcomes. Primary outcome (cardiac output) and secondary outcomes (stroke volume and mean arterial pressure) through the experiment. Lines are from linear regression models (with polynomials) where LBNP is treated as a continuous variable, giving the results presented in text and tables. Circles are estimations and error bars are 95% confidence intervals for each treatment at each LBNP level when treating LBNP levels as factors. BL, baseline; LBNP, lower body negative pressure.

There were no significant interaction effects for either methoxyflurane (−0.012 L min^−1^; 95% CI −0.060 to 0.036 L min^−1^; *P*=0.625) or fentanyl (0.039 L min^−1^; 95% CI −0.012 to 0.090 L min^−1^; *P*=0.134) on the linear effects of LBNP on cardiac output (Fig. 3 and Supplementary Table S1).

### Stroke volume and mean arterial pressure

There were no significant interaction effects for either methoxyflurane (0.26 ml; 95% CI −0.41 to 0.93 ml; *P*=0.447) or fentanyl (0.33 ml; 95% CI −0.37 to 1.0 ml; *P*=0.352) on the linear effects of LBNP on stroke volume (Fig. 3 and Supplementary Table S2).

There were also no significant interaction effects for either methoxyflurane (−0.25 mm Hg; 95% CI −0.74 to 0.25 mm Hg; *P*=0.321) or fentanyl (0.17 mm Hg; 95% CI −0.35 to 0.70 mm Hg; *P*=0.511) on the effects of LBNP on MAP (Fig. 3 and Supplementary Table S3).

### Time to decompensation

**Fig 4:**
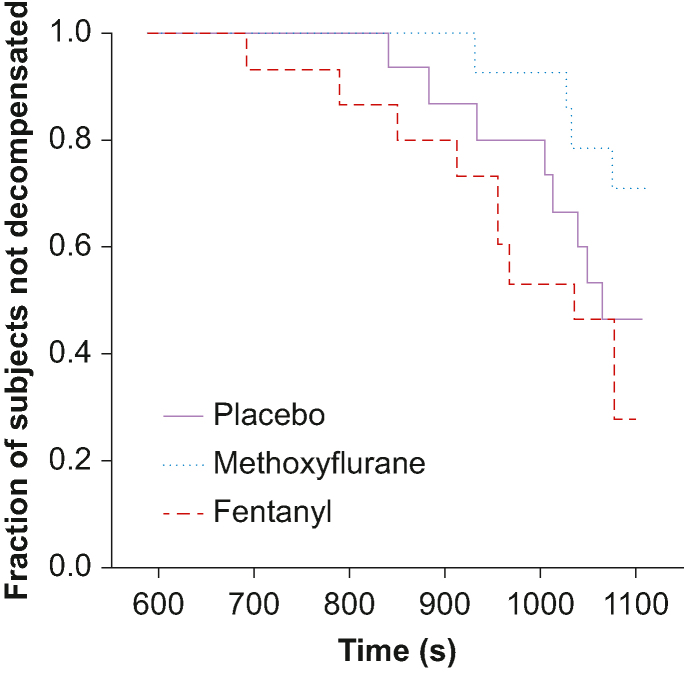
Time to haemodynamic decompensation. Kaplan–Meier plot of time to haemodynamic decompensation during LBNP for each treatment. Time starts at LBNP 0, giving 1080 s at completed LBNP 80. LBNP, lower body negative pressure.

In one subject, we experienced LBNP chamber failure at LBNP 50 with methoxyflurane, and data were censored from this level. Seven out of 15 subjects completed LBNP 80 with placebo, 10 out of 14 completed with methoxyflurane, and five out of 15 with fentanyl. The time to decompensation is presented in Fig. 4. There was no significant difference between methoxyflurane and placebo (hazard ratio [HR] −1.3; 95% CI −2.8 to 0.23; *P*=0.117) or fentanyl and placebo (HR 0.60; 95% CI −0.62 to 1.82; *P*=0.478). There was, however, a difference between fentanyl and methoxyflurane (HR 1.9; 95% CI 0.4–3.4; *P*=0.010).

### Other variables

**Fig 5:**
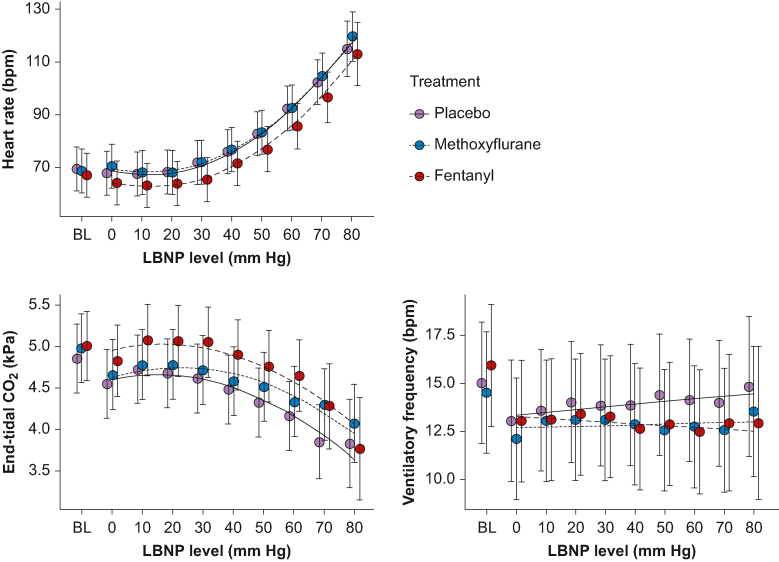
Heart rate, end-tidal CO_2_, and ventilatory frequency through the experiment. Lines are from linear regression models (with polynomials) where LBNP is treated as a continuous variable, giving the results presented in text and tables. Circles are estimations and error bars are 95% confidence intervals for each drug at each LBNP level when treating LBNP levels as factors. BL, baseline; LBNP, lower body negative pressure.

Heart rate, ETCO_2_, and ventilatory frequency data are presented in Fig. 5 and Supplementary Tables S4–S6. For ETCO_2_, there was a significant main effect of fentanyl (0.20 kPa; 95% CI 0.035–0.37 kPa; P=0.018, indicating a lower decrease from baseline to LBNP 0 compared with placebo. There was also a significant interaction effect with methoxyflurane (0.038 kPa; 95% CI 0.0023–0.074 kPa; *P*=0.037), indicating less reduction with increasing LBNP compared with placebo. For the other variables, there were no significant main or interaction effects of drugs. SpO_2_, ScerO_2_, and Laser Doppler flowmetry data are presented in Supplementary Figs. S1–S3 and Supplementary Tables S7–S8.

### Symptoms

**Fig 6:**
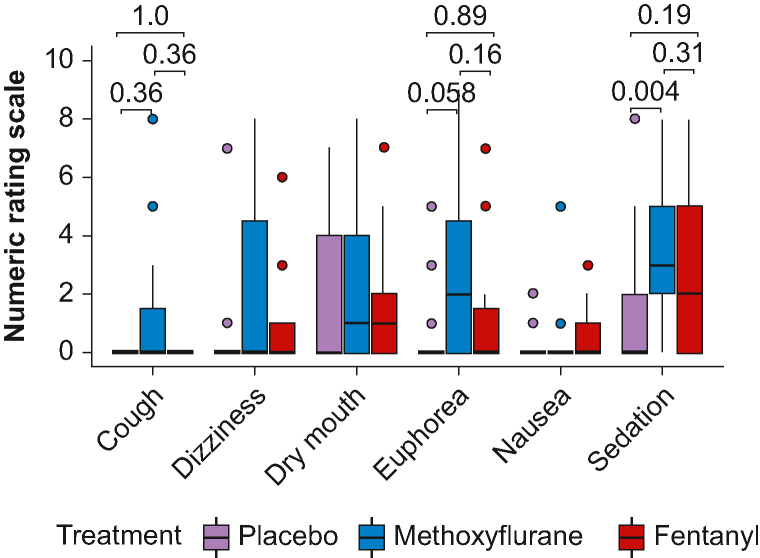
Symptoms during LBNP for each treatment. Boxes between first and third quartiles with medians marked. Whiskers to 1.5×inter-quartile range. For significant overall Friedman tests, *P*-values for pairwise *post hoc* comparisons (single-step corrections) are presented. LBNP, lower body negative pressure.

No subjects reported headache, confusion, visual disturbance, or muscular rigidity (VNRS=0). Further symptoms are presented in Fig. 6. For the overall Friedman test, there was an effect of drug on cough ($\chi^{2}$ =10.0; degrees of freedom [df]=2; *P*=0.007), euphoria ($\chi^{2}$ =10.0; df=2; *P*=0.007), and sedation ($\chi^{2}$ =13.1; df=2; *P*=0.001). For *post hoc* pairwise comparisons, there was only a significant difference between methoxyflurane and placebo for sedation (*P*=0.004).

## Discussion

The main finding of this study was that there was no effect of methoxyflurane on cardiac output compared with placebo during experimental hypovolaemia in the LBNP model. We also found no effect on stroke volume or MAP. Furthermore, we found no difference in time to decompensation between methoxyflurane or fentanyl and placebo, but there was a longer time to decompensation for methoxyflurane compared with fentanyl.

When used for general anaesthesia, methoxyflurane has been found to decrease cardiac output, MAP, and systemic vascular resistance, and increase the heart rate.^31^ Another study did not find a decrease in cardiac output, but an initial decrease in myocardial contractility.^32^ When used for analgesia, the doses are smaller and there do not seem to be significant adverse haemodynamic effects during prehospital use,^33^^,^^34^ in minor trauma in the emergency department^9^ or during bone marrow biopsies.^35^ Methoxyflurane is licensed for use in patients experiencing moderate to severe pain after trauma, and clinically evident cardiovascular instability is considered a contraindication.^8^ Diagnosing hypovolaemia in trauma patients is, however, no trivial task,^36^ but the present study indicates that methoxyflurane may be a safe alternative in patients with potential bleeding.

I.V. morphine, but not ketamine or fentanyl,^12^^,^^13^^,^^37^ has been found to reduce tolerance to experimental hypovolaemia in the LBNP model. Our findings for fentanyl are in line with the previous study,^12^ although the dose we used was considerably smaller.

We found a significant increase in sedation with methoxyflurane compared with placebo, consistent with our previous findings.^16^ Furthermore, self-administration using the inhaler may provide a degree of distraction and a sense of control which may be advantageous in trauma patients.

ScerO_2_ was reduced with increasing LBNP (Supplementary Table S7, Supplementary Fig. S2), as previously demonstrated.^24^^,^^38^ For methoxyflurane compared with placebo, ScerO_2_ initially increased from baseline to LBNP 0 and decreased thereafter. Whether this was a spurious effect remains to be elucidated, but there did not seem to be an effect of methoxyflurane beyond LBNP 10, further supporting the lack of haemodynamic effects of methoxyflurane during hypovolaemia. Acral laser Doppler flowmetry increased from baseline to LBNP 0 for fentanyl compared with placebo (Supplementary Table S8, Supplementary Fig. S3), indicating peripheral vasodilation.

There was a marginally lower minimal SpO_2_ for fentanyl compared with placebo (Supplementary Fig. S1). For methoxyflurane, we found no such reduction, and no effect on ventilatory frequency. These findings indicate a lack of adverse respiratory effects of methoxyflurane.

### Methodological considerations

The study was powered to detect a drug effect on cardiac output of 15%. However, several assumptions were incorporated into the simulation model. To avoid excessive complexity in this simulation, we did not incorporate the possibility of haemodynamic decompensation, which would result in fewer observations within some subjects. Based on our results, the estimates for methoxyflurane indicated a 95% confidence interval of −0.29 to 0.48 L min^−1^ at LBNP 80, corresponding to −5.4 to 9% of baseline values, indicating that the sample size was adequate based on the assumptions made. Further, the lower bound of −5.4% is probably of little clinical relevance, supporting a lack of adverse haemodynamic effects of 3 ml methoxyflurane. We chose cardiac output as our primary outcome, as it is proportional to global oxygen delivery assuming an unchanged haemoglobin concentration and arterial oxygen saturation.

Compared with a previous study^12^ and usual clinical practice, we administered a low dose of the comparator fentanyl, as 25 μg was the dose found comparable to 3 ml methoxyflurane in our previous study.^16^ In future studies of analgesics in hypovolaemia, one could consider adding pain to the model, in order to both increase doses of analgesics, and also increase validity. Analgesics are generally not given to patients without pain, and the haemodynamic effects in patients experiencing pain may differ, which is a limitation to our study. The subjects self-administered the methoxyflurane dose by breathing normally through the inhaler. However, we cannot guarantee that the dose was exactly the same for all subjects. Although every attempt was made to blind the subjects to the treatment allocation, experienced drug effects are impossible to avoid which could also potentially be perceived by the study personnel present.

The mean values for each LBNP level were trimmed to remove erroneous and outlying heartbeats generated by, for example, motion artifacts and the autocalibration of the Nexfin device. We chose to remove such outlying values by trimming, which is an objective method, as manual cleaning of the data would have introduced a subjective assessment of each value. Generally, with increased trimming, the trimmed mean approaches the median. The degree of trimming of 10% was largely arbitrarily chosen but gave values that seemed to represent the data well when plotted with the original beat-by-beat data for visual inspection. Further, analyses with 0%, 5%, and 20% trimming gave the same conclusion for the primary outcome.

In Fig 3, Fig 5, the estimates and confidence intervals for each LBNP level are from mixed model regression analyses, and not only calculated from the observations at each LBNP level. This was done to prevent the estimates being biased at higher LBNP levels as decompensated subjects no longer contributed data.

There may be group differences to the response to LBNP (e.g. between males and females).^39^ As this was a crossover study with statistical analyses using mixed models with subjects as a random effect, any such group differences in the response to LBNP alone should not influence the results. The mixed models are also useful for handling missing values, as occurred at lower LBNP values as a result of decompensation. Further, although the inclusion criteria for age were quite wide, the subjects included were all, except for one, in their twenties. This affects the generalisability of the results, which should be interpreted with caution in older adults and subjects with comorbidities. The randomised crossover design should also account for any systematic difference from the first to the third LBNP exposure.^40^ Other criteria to define decompensation than those in the present study have been used (e.g. a systolic blood pressure reduction <80 mm Hg). We chose MAP as our pressure criterion, as MAP determines perfusion. Further, compared with an absolute reduction, we chose a relative reduction because of the large interindividual variability of blood pressure.^41^ However, most often symptoms and the blood pressure thresholds coincide, and all sequences that were aborted with an MAP reduction in the present study also had a reduction in systolic blood pressure to <80 mm Hg. We did not continue LBNP exposure until decompensation in all subjects, the main reason being that cardiac output was chosen as our primary endpoint because of its central role in oxygen delivery. Further, the effect of both fentanyl and methoxyflurane was expected to taper off and play less of a role towards the end of the LBNP exposure, and even less if this were to be extended until decompensation. That being said, although the treatment effect was presumably less towards the end of the LBNP exposure of the present study, we believe some effect was present, as we found significant analgesic effects of both methoxyflurane and fentanyl 20 min after drug delivery in our previous study.^16^

Rating by VNRS is predominantly used for pain, but because of the familiarity with this rating system, we used it for all the symptoms. Although being studied for nausea,^42^ the validity of the VNRS results is questionable.

Haemodynamic variables may display circadian variations.^43^ Logistical limitations prevented us from standardising the time of day for the experiments. Six subjects had maximal differences in time of day of visits of more >5 h, but these did not seem to constitute outliers for the primary endpoint. Further, the time of day of the visit did not have a statistically significant effect on the primary outcome. The inhalers were prepared earlier on the day of the experiments. In a previous study, the loss of methoxyflurane was 5% after 25 h at 21°C in a low-density polyethylene bag.^44^

In summary, the present study does not indicate that methoxyflurane has significant adverse haemodynamic effects in conscious adults experiencing hypovolaemia. Future experimental studies could investigate repeated doses, possibly also with concurrent pain to increase the validity for clinical use.

## Authors' contributions

Study design: LØH, JH, MC, LAR, HL

Recruitment of volunteer participants: LØH, LOF, JH, HL

Data collection: LØH, LOF, JH, MC, HL

Data analysis: LØH, HL

Manuscript draft: LØH

Manuscript preparation and approval: all authors
