# Supplementary material for: Haemodynamic effects of methoxyflurane versus fentanyl and placebo in hypovolaemia: a randomised, double-blind crossover study in healthy volunteers
Source: BJA Open. 2023 Jun 28;7:100204. doi: 10.1016/j.bjao.2023.100204 (PMC10457468; doi:10.1016/j.bjao.2023.100204)
Supplement: Multimedia component 1 [file mmc1.docx]

### Supplementary Material

## Haemodynamic effects of methoxyflurane vs. fentanyl and placebo in hypovolaemia: A randomised, double-blind crossover study in healthy volunteers

### Regression models

In the tables, the final regression models are presented with the *changes from baseline* of the different outcome variables regressed on the predictors.

For the predictors, the treatment groups (placebo, methoxyflurane and fentanyl) are factors with placebo as the reference group. LBNP-level is a continuous variable ranging from 0 (LBNP 0 mmHg) to 8 (LBNP 80 mmHg). The effect of one unit LBNP change thus corresponds to a 10 mmHg LBNP change. Polynomials are designated with “^”, and interaction terms with “:”.

### Interpretation

The *Intercept* corresponds to the change from baseline to LBNP 0 for placebo, whereas the main effects of methoxyflurane and fentanyl correspond to the additional changes from baseline to LBNP 0 with each treatment. The *main effect* of LBNP-level and its polynomials (if statistically significant and included in the final model) describe the effect of LBNP on the outcome variable. The *interaction effects* between treatments and LBNP describe the additional effects of treatments on the effect of LBNP. The 1^st^ degree of LBNP and treatments with interaction effects are always included as a minimal final model.

## Cardiac output

**Supplementary Table 1.** Cardiac output (l min^-1^)

|  | Estimate | Std.Error | *P*-value |
| --- | --- | --- | --- |
| Intercept | −0.143 | 0.131 | 0.275 |
| LBNP-level | −0.195 | 0.0800 | 0.0153 |
| Methoxyflurane | −0.0587 | 0.109 | 0.592 |
| Fentanyl | −0.214 | 0.111 | 0.0544 |
| LBNP-level^2 | −0.0490 | 0.0245 | 0.0459 |
| LBNP-level^3 | 0.00476 | 0.00207 | 0.0223 |
| LBNP-level:Methoxyflurane | −0.0120 | 0.0246 | 0.625 |
| LBNP-level:Fentanyl | 0.0390 | 0.0260 | 0.134 |

## Stroke volume

**Supplementary Table 2.** Stroke volume (ml)

|  | Estimate | Std.Error | *P*-value |
| --- | --- | --- | --- |
| Intercept | −1.24 | 2.46 | 0.614 |
| LBNP-level | −0.663 | 1.11 | 0.549 |
| Methoxyflurane | −3.12 | 1.51 | 0.0398 |
| Fentanyl | −0.645 | 1.53 | 0.674 |
| LBNP-level^2 | −1.96 | 0.338 | 1.6 × 10^−8^ |
| LBNP-level^3 | 0.154 | 0.0287 | 1.3 × 10^−7^ |
| LBNP-level:Methoxyflurane | 0.259 | 0.340 | 0.447 |
| LBNP-level:Fentanyl | 0.334 | 0.359 | 0.352 |

## Mean arterial pressure

**Supplementary Table 3.** Mean arterial pressure (mmHg)

|  | Estimate | Std.Error | P-value |
| --- | --- | --- | --- |
| Intercept | −0.972 | 1.12 | 0.386 |
| LBNP-level | 1.10 | 0.818 | 0.180 |
| Methoxyflurane | 2.09 | 1.12 | 0.0627 |
| Fentanyl | −0.133 | 1.13 | 0.906 |
| LBNP-level^2 | −0.522 | 0.250 | 0.0376 |
| LBNP-level^3 | 0.0447 | 0.0212 | 0.0358 |
| LBNP-level:Methoxyflurane | −0.250 | 0.252 | 0.321 |
| LBNP-level:Fentanyl | 0.175 | 0.266 | 0.511 |

## Heart rate

**Supplementary Table 4.** Heart rate (beats min^-1^)

|  | Estimate | Std.Error | P-value |
| --- | --- | --- | --- |
| Intercept | −0.759 | 1.64 | 0.645 |
| LBNP-level | −2.44 | 0.528 | 5.5 × 10^−6^ |
| Methoxyflurane | 1.97 | 1.50 | 0.189 |
| Fentanyl | −1.75 | 1.52 | 0.249 |
| LBNP-level^2 | 1.06 | 0.0625 | 1.8 × 10^−47^ |
| LBNP-level:Methoxyflurane | −0.0597 | 0.337 | 0.859 |
| LBNP-level:Fentanyl | −0.326 | 0.356 | 0.359 |

## End-tidal CO_2_

**Supplementary Table 5.** End-tidal CO_2_ (kPa)

|  | Estimate | Std.Error | P-value |
| --- | --- | --- | --- |
| Intercept | −0.249 | 0.101 | 0.0139 |
| LBNP-level | 0.0732 | 0.0286 | 0.0109 |
| Methoxyflurane | −0.111 | 0.0814 | 0.175 |
| Fentanyl | 0.200 | 0.0839 | 0.0175 |
| LBNP-level^2 | −0.0244 | 0.00339 | 4.3 × 10^−12^ |
| LBNP-level:Methoxyflurane | 0.0381 | 0.0182 | 0.0370 |
| LBNP-level:Fentanyl | 0.00878 | 0.0196 | 0.654 |

## Respiratory rate

**Supplementary Table 6.** Respiratory rate (breaths min^-1^)

|  | Estimate | Std.Error | P-value |
| --- | --- | --- | --- |
| Intercept | −1.62 | 0.795 | 0.0421 |
| LBNP-level | 0.136 | 0.108 | 0.210 |
| Methoxyflurane | −0.224 | 0.664 | 0.736 |
| Fentanyl | −1.02 | 0.685 | 0.136 |
| LBNP-level:Methoxyflurane | −0.0923 | 0.149 | 0.537 |
| LBNP-level:Fentanyl | −0.233 | 0.161 | 0.148 |

## SpO_2_

Due to the ceiling of 100% for SpO_2_, a regression analysis on all the observations was not performed, and data are presented as boxplots. The minimal values within each subject for each treatment were: placebo: 97 % (96, 98 (median (25th, 75th percentiles)); methoxyflurane 97 % (96, 99) and fentanyl 96 % (95, 97). These were compared using a Friedman test giving a significant effect of treatment (χ2=12.1; df=2; *P*=0.0023). The Nemenyi post-hoc test showed a lower minimal value for fentanyl compared to placebo (*P*=0.0015).


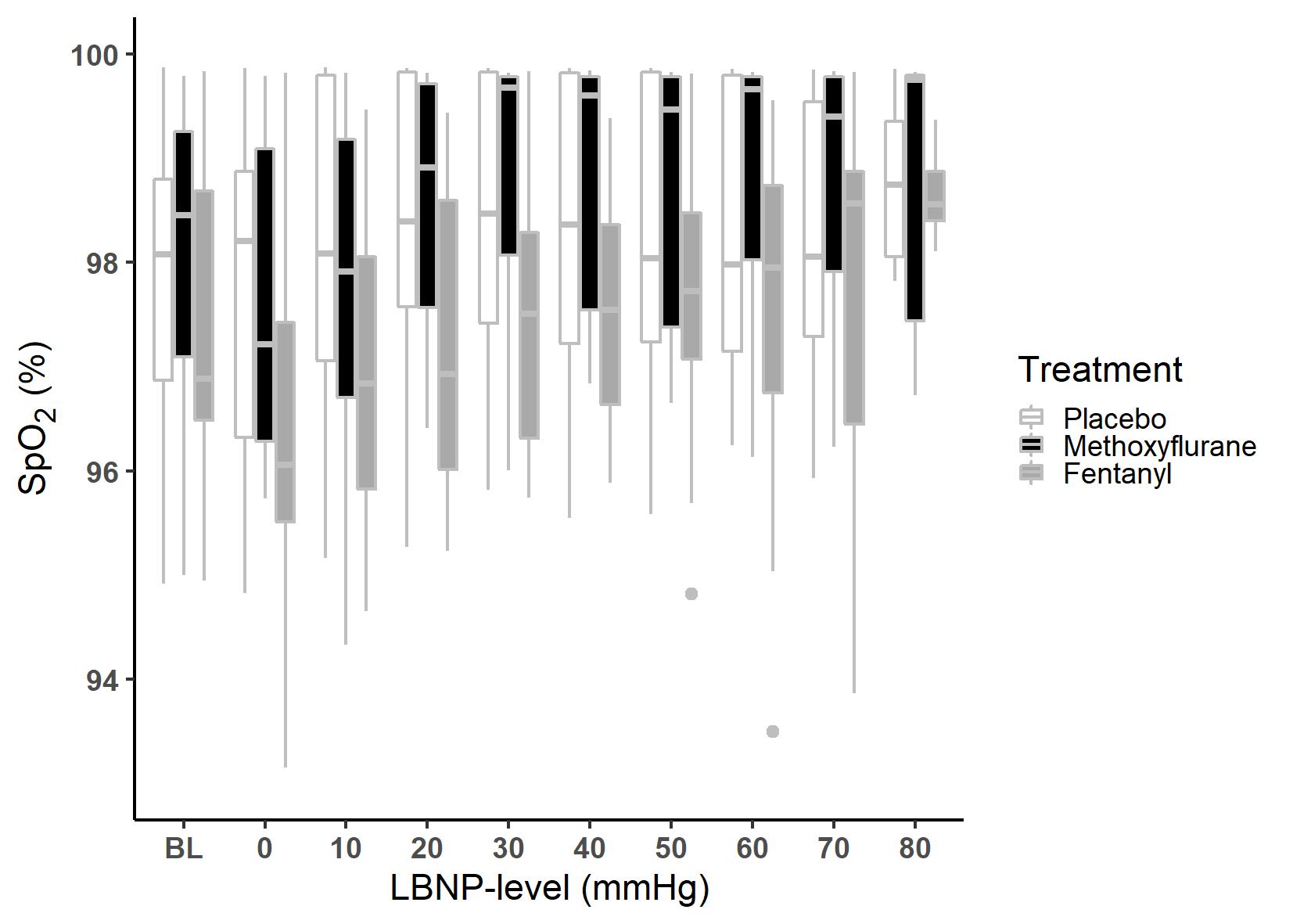


**Supplementary Fig 1.** Arterial oxygen saturation (SpO_2_) through the experiment. Boxes between first and third quartiles with medians marked. Whiskers to 1.5 × interquartile range. LBNP, lower body negative pressure; BL, baseline.

## Cerebral oximetry, ScerO_2_

**Supplementary Table 7.** Cerebral oxygen saturation (%)

|  | Estimate | Std.Error | P-value |
| --- | --- | --- | --- |
| Intercept | −0.847 | 0.645 | 0.190 |
| LBNP-level | −1.06 | 0.443 | 0.0172 |
| Methoxyflurane | 2.52 | 0.685 | 2.7 × 10^−4^ |
| Fentanyl | 0.953 | 0.675 | 0.159 |
| LBNP-level^2 | 0.195 | 0.121 | 0.107 |
| LBNP-level^3 | −0.0204 | 0.00992 | 0.0404 |
| LBNP-level:Methoxyflurane | −1.10 | 0.414 | 0.00848 |
| LBNP-level:Fentanyl | 0.602 | 0.418 | 0.151 |
| Methoxyflurane:LBNP-level^2 | 0.113 | 0.0521 | 0.0303 |
| Fentanyl:LBNP-level^2 | −0.103 | 0.0542 | 0.0571 |


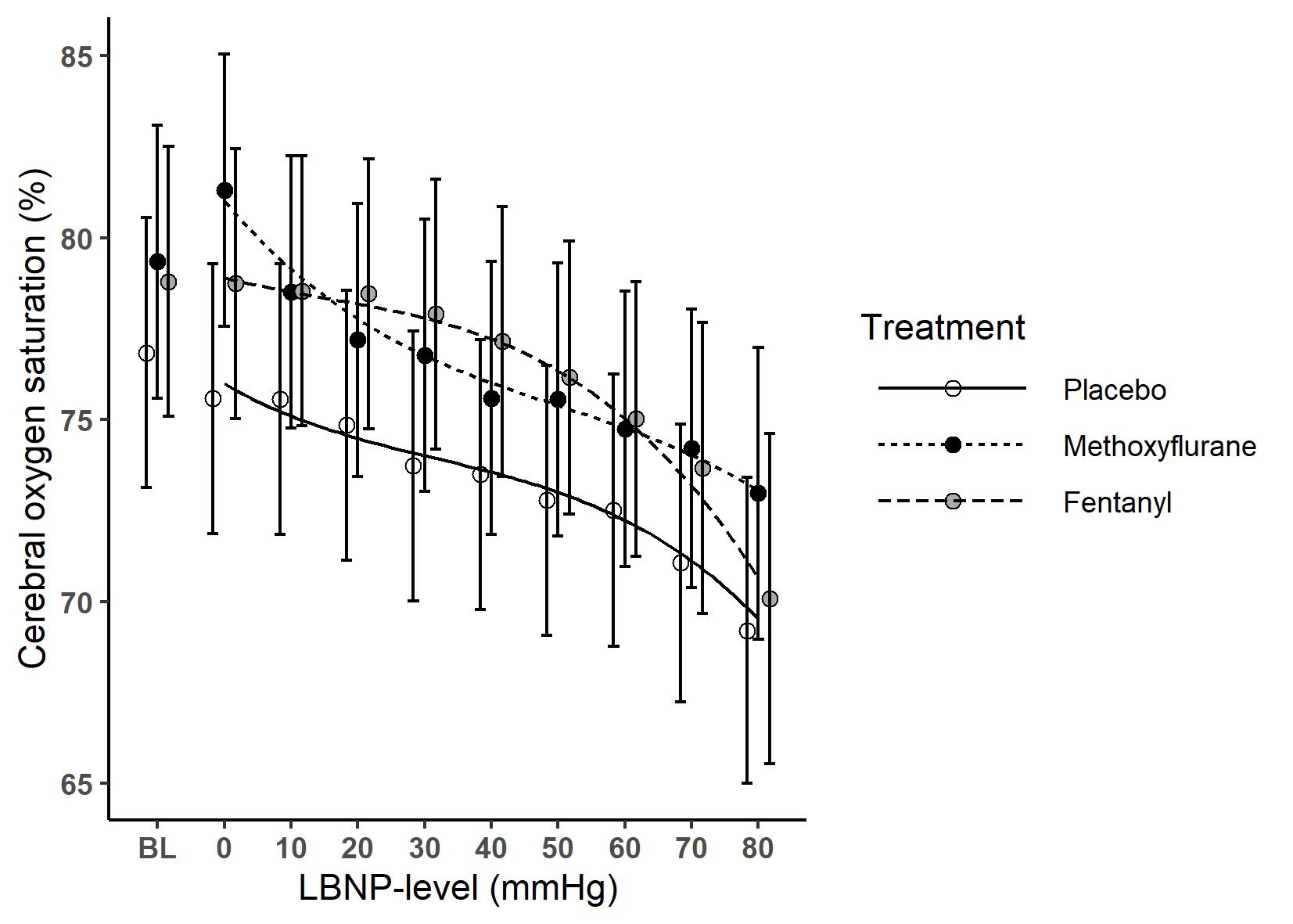


**Supplementary Fig 2**. Cerebral oxygen saturation (%). LBNP, lower body negative pressure; BL, baseline.

## Laser Doppler flowmetry

**Supplementary Table 8.** Laser Doppler flowmetry [log(AU)]. AU, arbitrary units

|  | Estimate | Std.Error | P-value |
| --- | --- | --- | --- |
| Intercept | −0.213 | 0.182 | 0.243 |
| LBNP-level | −0.0273 | 0.0285 | 0.339 |
| Methoxyflurane | 0.350 | 0.179 | 0.0514 |
| Fentanyl | 0.457 | 0.177 | 0.0104 |
| LBNP-level:Methoxyflurane | −0.0359 | 0.0402 | 0.372 |
| LBNP-level:Fentanyl | −0.0402 | 0.0418 | 0.338 |


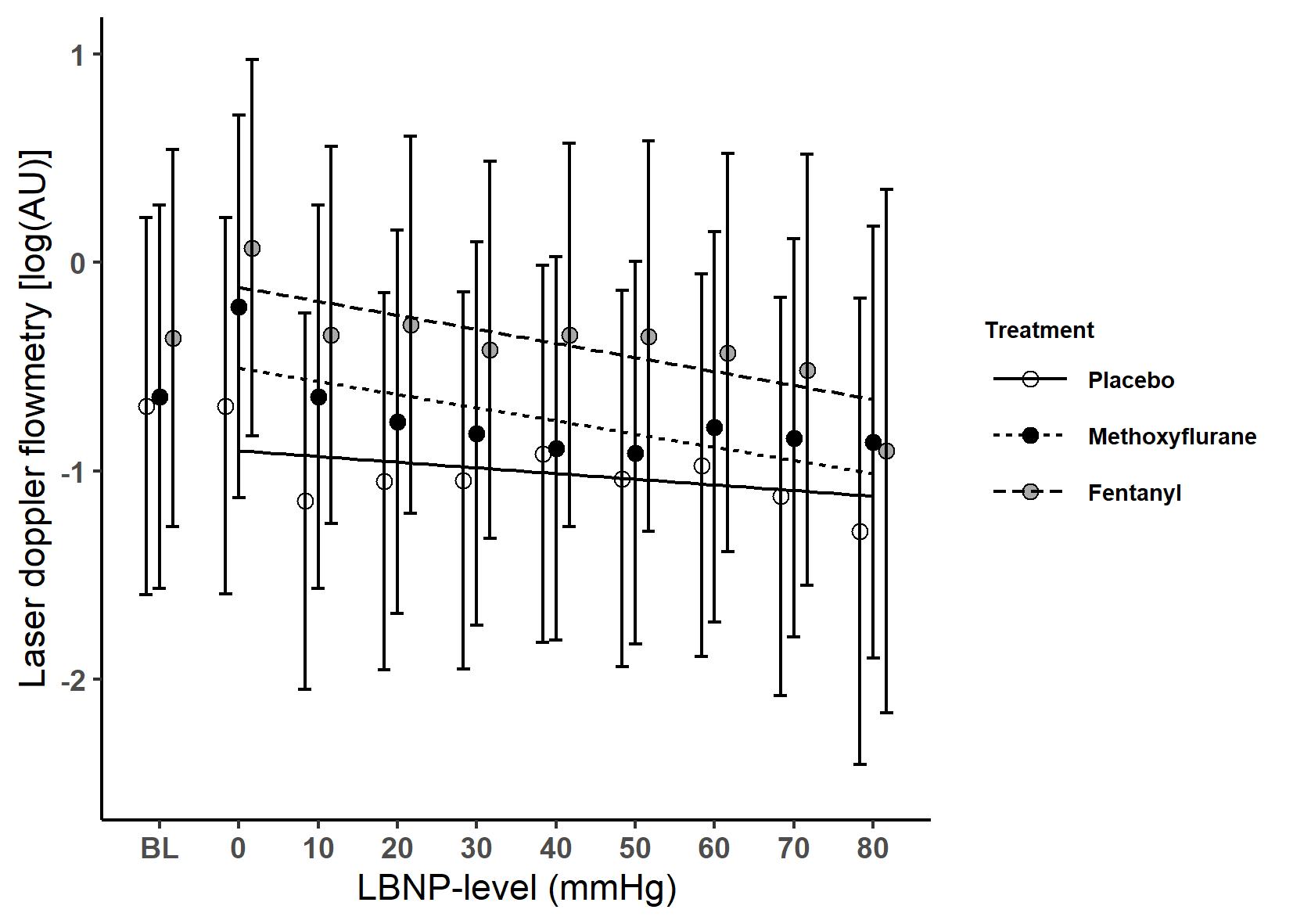


**Supplementary Fig 3.** Laser Doppler flowmetry [log(AU)]. LBNP, lower body negative pressure; BL, baseline.
